# Contrast induced nephropathy has to be differentiated from kidney injury due to atheroembolic disease

**DOI:** 10.12861/jrip.2013.34

**Published:** 2013-09-01

**Authors:** Hamid Nasri, Muhammed Mubarak

**Affiliations:** ^1^Department of Nephrology, Division of Nephropathology, Isfahan University of Medical Sciences, Isfahan, Iran; ^2^Department of Histopathology, Sindh Institute of Urology and Transplantation (SIUT), Karachi, Pakistan

**Keywords:** Contrast induced nephropathy, Acute kidney injury, Atheroembolic disease

**Implication for health policy/practice/research/medical education:**

Atheroembolic renal disease is often overlooked as a cause of kidney injury. This diagnosis should be strongly considered in any patient with kidney damage and high risk factors for atherosclerotic disease. Renal biopsy can be of diagnostic value and the nephropathologists should pay careful attention to vessels on such biopsies, as the lesions can be focal and can be missed.

## Case presentation

An 85-year-old man was referred to the hospital for the evaluation of renal failure. Patient had the complaint of edema of lower extremities, dyspnea on exertion, skin lesions, nausea and vomiting. He also complained of orthopnea and cough. In the past medical history, he had the history of diabetes, hypertension and coronary artery disease (CAD). He had undergone coronary angiography three months before admission. Patient’s complaints gradually increased after angiography. The patient was admitted with blood pressure of 100/80 mmHg and crackles in the base of the lungs. There was livedo reticularis on the feet (Figure 1A). His initial investigations showed hemoglobin of 8 g/dl and serum creatinine of 10 mg/dl. The urinalysis was bland. Ejection fraction on Doppler echocardiography was 25-30%, and pulmonary artery pressure was 75 mmHg. Given the history of angiography and the onset of symptoms following the procedure, a clinical diagnosis of contrast induced kidney injury was contemplated.

Figure 1 
The clinical and morphological features seen on the renal biopsy. A) The toes show bluish mottling of the skin consistent with livedo reticularis. B) A representative glomerulus showing essentially minor changes on light microscopy. The capillary lumina are patent, mesangial cellularity is within normal limits and Bowman’s space does not contain crescents (Jones methenamine silver stain, ×400). C) Silver stain showing mild tubular atrophy, and interstitial fibrosis. (Jones methenamine silver stain, ×400). D) Medium-power view showing a small arcuate artery with luminal occlusion, peri-adventitial inflammation and fibrosis. (Silver-Periodic acid-Schiff (PAS), ×200).

**A:**
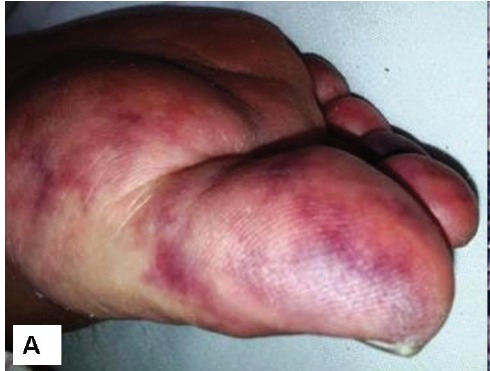

**B:**
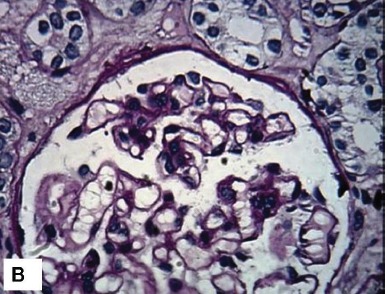

**C:**
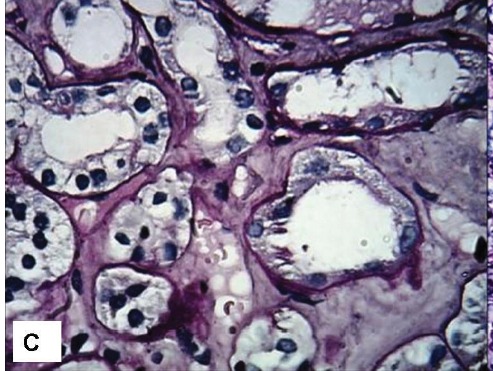

**D:**
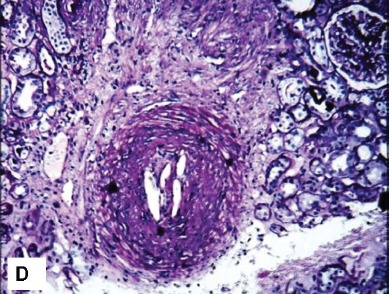

A temporary jugular access was inserted and hemodialysis was started. After five dialysis sessions and improvement of his condition, the patient was scheduled for renal biopsy. In the semi-recumbent position, a kidney biopsy was performed. On the renal biopsy, glomeruli had normal morphology and architecture (Figure 1B). Interstitium exhibited edema, infiltration, fibrosis and tubular atrophy (~ 30%) (Figure 1C). The tubular epithelial cells showed marked degenerative changes and there was tubular dilatation. Interlobular and arcuate arteries showed a peri-vascular infiltration, fibrosis and luminal occlusion. The lumina showed prominent needle-like clefts and mild inflammatory cell infiltration (Figure 1D). Immunofluorescence (IF) studies for IgA, IgG, IgM, C3, C1q and fibrin were negative.

The diagnosis was entirely consistent with atheroembolic disease, hence patient’s supportive care and dialysis was continued. Two months after discharge, the patient died at home.

## Discussion

**Figure 2:**
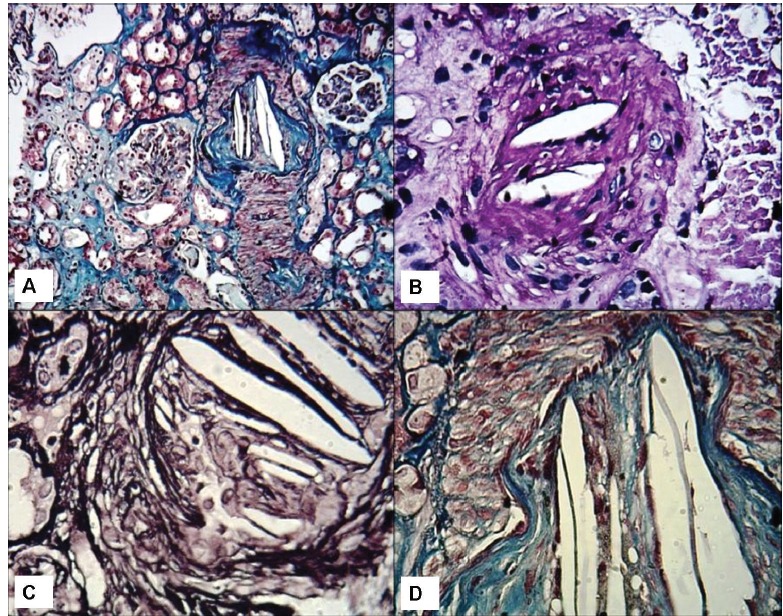
The morphological features of atheroembolic vascular disease of the kidney in greater detail. A) Low-power view showing one interlobular artery with luminal occlusion and needle-like crystals in the lumina. Two glomeruli are seen in the vicinity with minor changes on light microscopy. Mild patchy tubular atrophy is seen. (Masson’s trichrome stain, ×100). B) Medium-power view showing one small artery with luminal cholesterol clefts causing luminal occlusion (Period acid-Schiff stain, ×200). C) High-power view showing typical empty-looking and needle-like cholesterol clefts in the lumina (Jones methenamine silver stain, ×400). D) High-power view showing luminal cholesterol clefts and intimal fibrosis. Media appears red on this stain. (Masson’s trichrome stain, ×400).

Fenger, a Danish physician and his colleagues, described this disease for the first time in 1844 (1). The disease is the result of showers of cholesterol crystals released by ruptured atherosclerotic plaques (1-3). The disease may occur by spontaneous embolization or after angiographic procedures. The syndrome of cholesterol embolization has various clinical features (2-4). It may lead to acute, sub-acute or chronic kidney injury, skin emboli or gut ischemia. Skin manifestations which are most commonly livedo reticularis and blue toe syndrome are usually limited to the lower extremities (1-5). In fact, cholesterol crystals trigger an inflammatory reaction after they lodge in the small arteries of the target organs including kidneys (3-6). Various constitutional symptoms and signs consisting of fever, anorexia, fatigue, weight loss and myalgia are usually the manifestations of the inflammatory response (4-7). Kidney biopsy seems to be a reliable diagnostic tool in patients with atheroembolic disease. However, the typical lesion, such as blockage of small arteries, arterioles, and the glomerular capillaries, usually are focal, and can be easily overlooked, if not carefully looked for. The classic lesion in atheroembolic disease is the occlusion of interlobular /arcuate arteries, small arteries, arterioles and the glomerular capillaries with cholesterol emboli (2-5). The emboli of cholesterol crystals generally are defined by the characteristic biconvex, needle-shaped clefts appearing as “ghosts” (Figure 2 A-D). The crystals normally are dissolved during routine histologic preparation (3-7). The prognosis in this disease is generally considered to be very poor. The reported mortality varies from 64 to 81% (4-9), depending on a number of factors, such as age and cardiovascular status. A variety of therapeutic modalities are used with conflicting results on their beneficial effects. A focus on early diagnosis and more efficient therapeutic and preventive strategies are therefore needed.

## Authors’ contribution

MM and HN wrote the manuscript equally.

## Conflict of interests

The author declared no competing interests.

## Ethical considerations

Ethical issues (including plagiarism, misconduct, data fabrication, falsification, double publication or submission, redundancy) have been completely observed by the author.

## Funding/Support

None.
